# Genome Sequence of *Streptomyces viridosporus* Strain T7A ATCC 39115, a Lignin-Degrading Actinomycete

**DOI:** 10.1128/genomeA.00416-13

**Published:** 2013-07-05

**Authors:** Jennifer R. Davis, Lynne Goodwin, Hazuki Teshima, Chris Detter, Roxanne Tapia, Cliff Han, Marcel Huntemann, Chia-Lin Wei, James Han, Amy Chen, Nikos Kyrpides, Kostas Mavrommatis, Ernest Szeto, Victor Markowitz, Natalia Ivanova, Natalia Mikhailova, Galina Ovchinnikova, Ioanna Pagani, Amrita Pati, Tanja Woyke, Sam Pitluck, Lin Peters, Matt Nolan, Miriam Land, Jason K. Sello

**Affiliations:** Department of Molecular Pharmacology, Physiology and Biotechnology, Brown University, Providence, Rhode Island, USAa; Los Alamos National Laboratory, Bioscience Division, Los Alamos, New Mexico, USAb; DOE Joint Genome Institute, Walnut Creek, California, USAc; Oak Ridge National Laboratory, Oak Ridge, Tennessee, USAd; Department of Chemistry, Brown University, Providence, Rhode Island, USAe

## Abstract

We announce the availability of the genome sequence of *Streptomyces viridosporus* strain T7A ATCC 39115, a plant biomass-degrading actinomycete. This bacterium is of special interest because of its capacity to degrade lignin, an underutilized component of plants in the context of bioenergy. It has a full complement of genes for plant biomass catabolism.

## GENOME ANNOUNCEMENT

In the search for fossil fuel alternatives, much effort has been devoted to the discovery and development of microorganisms that can efficiently convert plant biomass into biofuels and commodity chemicals (1, 2). Many microorganisms have been identified that can degrade the carbohydrate components in plants. However, few are known that also depolymerize and consume lignin, a structural polymer in plant cell walls. One such organism is *Streptomyces viridosporus* strain T7A ATCC 39115, a soil-dwelling actinomycete (3–7). Importantly, it is the source of the first bacterial peroxidase that depolymerizes lignin (8–12). The absence of information on the genes encoding this lignin peroxidase and other enzymes responsible for lignin degradation and their biotechnological potential motivated our efforts to sequence the *S. viridosporus* genome.

The draft genome sequence of *S. viridosporus* was generated at the Department of Energy (DOE) Joint Genome Institute (JGI) using a combination of Illumina (13) and 454 technologies (14). The Illumina GAii shotgun library generated 67,837,180 reads totaling 5,155.6 Mb. The 454 Titanium standard library generated 228,388 reads, and the paired-end 454 library (with an average insert size of 8 kb) generated 635,872 reads, totaling 223.4 Mb of 454 data. The 454 data were assembled together with Newbler, version 2.3, while Illumina sequencing data were assembled with Velvet, version 1.0.13 (15). The 454 and Illumina assemblies were integrated using parallel Phrap, version SPS-4.24 (High Performance Software, LLC). Illumina data were used to correct potential base errors and increase consensus quality using the software Polisher developed at the JGI (A. Lapidus, unpublished). Possible misassemblies were corrected using gapResolution (C. Han, unpublished) or Dupfinisher (16), or sequencing cloned bridging PCR fragments with subcloning. Gaps between contigs were closed by editing in Consed (17–19), by PCR, and by bubble PCR (J.-F. Cheng, unpublished) primer walks. A total of 442 additional reactions were necessary to close gaps and to raise the quality of the finished sequence. The estimated genome size is 8.3 Mb and the final assembly is based on 163.6 Mb of 454 draft data, which provide an average 19.7× coverage of the genome, and 5,006.8 Mb of Illumina draft data, which provide an average 603.2× coverage of the genome.

The total genome size is 8,278,598 bp with a G+C content of 72.5%. Prodigal software (20) and the JGI GenePRIMP pipeline (21) were used to identify 7,552 candidate protein-encoding genes. Annotations using the NCBI nonredundant database, UniProt, TIGRFam, Pfam, Priam, KEGG, COG, and InterPro databases were completed and the results can be accessed at http://img.jgi.doe.gov.

The genome contains numerous genes encoding homologs of enzymes that deconstruct plant biomass. COG annotation showed that 8.35% of the predicted proteins are involved in carbohydrate transport and metabolism. Genes encoding putative lignin-degrading enzymes, such as heme peroxidases, Dyp-type peroxidases, and catalases, were identified and are currently being analyzed. Pathways for the catabolism of lignin-derived aromatic compounds, such as protocatechuate, benzoate, and catechol, were also identified. Based on these findings, we anticipate that *S. virdosporus* or its complement of genes for plant catabolism could constitute a lignocellulose biorefinery.

### Nucleotide sequence accession number.

The genome sequence of *Streptomyces viridosporus* has been deposited in GenBank under accession no. AJFD00000000.
